# Genome-Wide Transcriptome Analysis Reveals Extensive Alternative Splicing Events in the Protoscoleces of *Echinococcus granulosus* and *Echinococcus multilocularis*

**DOI:** 10.3389/fmicb.2017.00929

**Published:** 2017-05-23

**Authors:** Shuai Liu, Xiaosu Zhou, Lili Hao, Xianyu Piao, Nan Hou, Qijun Chen

**Affiliations:** ^1^MOH Key Laboratory of Systems Biology of Pathogens, Institute of Pathogen Biology, Chinese Academy of Medical Sciences and Peking Union Medical CollegeBeijing, China; ^2^College of Life Science and Technology, Southwest University for NationalitiesChengdu, China; ^3^Key Laboratory of Zoonosis, Shenyang Agriculture UniversityShenyang, China

**Keywords:** *Echinococcus granulosus*, *Echinococcus multilocularis*, transcriptome, alternative splicing, nextgeneration sequencing

## Abstract

Alternative splicing (AS), as one of the most important topics in the post-genomic era, has been extensively studied in numerous organisms. However, little is known about the prevalence and characteristics of AS in *Echinococcus* species, which can cause significant health problems to humans and domestic animals. Based on high-throughput RNA-sequencing data, we performed a genome-wide survey of AS in two major pathogens of echinococcosis*-Echinococcus granulosus* and *Echinococcus multilocularis*. Our study revealed that the prevalence and characteristics of AS in protoscoleces of the two parasites were generally consistent with each other. A total of 6,826 AS events from 3,774 *E. granulosus* genes and 6,644 AS events from 3,611 *E. multilocularis* genes were identified in protoscolex transcriptomes, indicating that 33–36% of genes were subject to AS in the two parasites. Strikingly, intron retention instead of exon skipping was the predominant type of AS in *Echinococcus* species. Moreover, analysis of the Kyoto Encyclopedia of Genes and Genomes pathway indicated that genes that underwent AS events were significantly enriched in multiple pathways mainly related to metabolism (e.g., purine, fatty acid, galactose, and glycerolipid metabolism), signal transduction (e.g., Jak-STAT, VEGF, Notch, and GnRH signaling pathways), and genetic information processing (e.g., RNA transport and mRNA surveillance pathways). The landscape of AS obtained in this study will not only facilitate future investigations on transcriptome complexity and AS regulation during the life cycle of *Echinococcus* species, but also provide an invaluable resource for future functional and evolutionary studies of AS in platyhelminth parasites.

## Introduction

Cystic echinococcosis and alveolar echinococcosis are potentially fatal chronic and progressive zoonoses affecting humans and domestic animals (Eckert and Deplazes, 2004; Craig and Larrieu, 2006). These diseases are caused by the metacestode larval stage of two related cestodes, *Echinococcus granulosus* and *Echinococcus multilocularis*. The life cycles of both of the parasitic cestodes require intermediate hosts (wild or domesticated ungulates, rodents, and humans are the intermediate hosts for larval parasites), and definitive hosts (domestic dogs, foxes, and other wild carnivores are the definitive hosts for adult parasites). The intermediate hosts are infected by the oral uptake of eggs, which contain oncospheres. Once the eggs are activated within the host’s digestive system, the oncospheres hatch from the eggs, penetrate the intestinal wall, and are carried by the bloodstream to the liver, lungs, or other organs, turning into hydatid cysts filled with fluid and protoscoleces. Protoscoleces, as the key infectious component of the larval stages, can interact with both definitive and intermediate hosts. They can asexually proliferate in intermediate hosts and mature into adult parasites when the hydatid cysts are swallowed by the definitive host (McManus et al., 2003). The parasites have exploited daedal strategies to adapt to the distinct environments of different hosts and to evade host immune responses, yet the concrete mechanisms of these strategies remain poorly understood.

Alternative splicing, as an essential characteristic of eukaryotic gene expression, is a post-transcriptional process that enables one gene to encode two or more mature mRNAs (Nilsen and Graveley, 2010). It plays a key role in the transmission of genetic information from DNA to proteins by expanding the coding capacity of genomes (Naftelberg et al., 2015). Alternative splice products may generate proteins with different structures, sub-cellular localizations, and molecular functions, some of which are putative targets for vaccines or drugs (Keren et al., 2010; Yeoh et al., 2015). Regulation of AS is as important as regulation of transcription to determine cell- and tissue-specific features, normal cell functioning, and responses of eukaryotic cells to external cues (Kornblihtt et al., 2013; Naftelberg et al., 2015). For the parasite, AS may play important roles in the host–parasite interplay through generating alternative isoforms with different functions (Hull and Dlamini, 2014). The discovery of AS giving rise to different isoforms of antigenic proteins seems to be indicative of a further means of immune evasion by the parasite (DeMarco et al., 2010; Hull and Dlamini, 2014).

As one of the next-generation sequencing approaches, RNA-Seq has been applied in numerous studies that have altered our view of the extent and complexity of eukaryotic transcriptomes (Wang et al., 2009; Marguerat and Bahler, 2010). This technique enables researchers to survey the entire transcriptome in a very high-throughput and quantitative manner, which provides inviting information about transcriptional and post-transcriptional gene regulation, and gives unique insight into the variety of transcript structures and processing on a global scale (Mortazavi et al., 2008; Sultan et al., 2008). In particular, RNA-Seq data can be directly used to identify AS events in transcriptomes by searching for sequence reads that span splice junctions, which has dramatically enriched our knowledge of AS in the eukaryotic kingdom (Martin and Wang, 2011; Kornblihtt et al., 2013; Staiger and Brown, 2013; Hull and Dlamini, 2014; Filichkin et al., 2015). Recently, the decoding and availability of the genome sequences of two *Echinococcus* species has proved pivotal for the systematic dissection of the biology of the parasites (Tsai et al., 2013; Zheng et al., 2013). The main obstacle lies in that a precise estimate of AS events cannot be obtained merely based on genomic sequence analysis. As far as we know, deep-sequencing research explicitly related to AS in *Echinococcus* species has not been conducted, even though a few studies focusing on transcriptome profile analyses of *E. granulosus* (Pan et al., 2014) or *E. multilocularis* (Huang et al., 2016) have been reported using next-generation sequencing approaches.

In the present study, we have investigated AS events in protoscolex transcriptomes of both *E. granulosus* and *E. multiloculari*s using a high-throughput RNA-Seq approach. We identified 1000s of AS events in the two parasites, which can be classified into seven splicing patterns. Interestingly, intron retention was found to be the predominant splicing type in protoscoleces. This provides a valuable basis for a more precise, dynamic understanding of the transcriptome in *Echinococcus* species.

## Materials and Methods

### Ethics Statement

All procedures performed on animals in this study were conducted following the animal husbandry guidelines of the Chinese Academy of Medical Sciences and with permission from the Experimental Animal Committee of the Chinese Academy of Medical Sciences with the Ethical Clearance Number IPB-2011-8.

### Parasites

All parasite materials were collected from the Xinjiang Uyghur Autonomous Region, China. For *E. granulosus* transcriptome sequencing, a unilocular cyst was isolated from a sheep liver infected with *E. granulosus*. After rinsing 10 times with normal saline, protoscoleces were aspirated from the hydatid cysts, washed three times with normal saline and then soaked in RNA*later* solution (Ambion, CA, United States); samples were stored at -80°C until total RNA was isolated. Hydatid cysts of *E. multilocularis* were freshly isolated from male gerbils at 5–6 months after infection with *E. multilocularis*. After rinsing with normal saline, the cysts were opened and homogenized to a smooth consistency in 200 ml normal saline. The suspension was successively passed through 40, 80, 140, and 325 mesh metal sieves. The protoscoleces on the 325 mesh metal sieve were collected into a beaker and were washed five times with ice-cold normal saline. The storage method was as above.

### RNA Extraction and Deep Sequencing of mRNA

For mRNA library construction and deep sequencing, total RNA was isolated using an RNeasy Mini kit (Qiagen, Hilden, Germany) according to the manufacturer’s instructions. Potential contaminating genomic DNA was removed from RNA samples using a Turbo DNA-free kit (Ambion, CA, United States). The quantity and integrity of the total RNA was assessed using an Agilent 2100 Bioanalyzer (Agilent Technologies, United States). Normalized starting quantities of total RNA were used to prepare two biological replicates of Illumina sequencing libraries for each species using a TruSeq^TM^ RNA sample preparation kit (Illumina, San Diego, CA, United States). Following the manufacturer’s instructions, libraries were constructed and sequenced using a paired-end read protocol with 100 bp of data collected per run on an Illumina HiSeq^TM^ 2000 instrument housed at MininGene Biotechnology Co. Ltd, Beijing, China. Data analyses and base calling were performed using the Illumina instrument software.

### mRNA Transcriptome Data Processing and Bioinformatic Analyses

A Perl program was written to remove low-quality reads (more than half of the bases had a quality value less than 5). The retained high-quality reads were mapped to the published genomes of *E. granulosus* (Tsai et al., 2013; Zheng et al., 2013) and *E. multilocularis* (Tsai et al., 2013) by HISAT (Kim et al., 2015), and then assembled with StringTie (Pertea et al., 2015) to construct unique transcript sequences, using the parameters: -g -b -u -o. AS events in a gene were detected by SUPPA (Alamancos et al., 2015). Cuffquant (Trapnell et al., 2010) was used to quantify the expression of genes and transcripts, and the raw expression values were normalized by Cuffnorm. The expression values were calculated for each sample based on the number of fragments per kilobase of exon per million reads mapped. InterPro domain information (Mulder et al., 2003) was annotated by InterProScan (release 53.0) (Zdobnov and Apweiler, 2001) and functional assignments mapped with GO (Harris et al., 2004). WEGO (v3.3) (Ye et al., 2006) was used for GO classification. To obtain an overview of the gene pathway networks, KEGG analysis was performed by comparing the transcripts with the KEGG database (release 58) (Kanehisa et al., 2006) using BLASTX (Altschul et al., 1997; Ouyang et al., 2007) at *E*-values ≤ 1E^-10^. A Perl script was used to retrieve KO information from the BLAST results, and then establish pathway associations between the transcripts and database. A Perl script was written to count the number of total genes and the number of AS genes in a certain functional pathway, then a significant score *P*-value was calculated using the R scripts under hypergeometric distribution. A function pathway with *P*-value < 0.05 was deemed as enrichment in AS genes.

### Verification of AS Events by RT-PCR

To validate AS events experimentally, RT-PCR was performed for eight pairs of homologous genes that underwent different AS types in the two parasites. For each sample, 1 μg total RNA was reverse transcribed into first-strand cDNA using a SuperScript III Reverse Transcriptase kit (Invitrogen, Carlsbad, CA, United States) with oligo(dT)_12-18_ primer. Primers were designed based on the consensus gene sequences of the two parasites, and the primer sequences are listed in Supplementary Table S1. PCR was performed in a 25 μl reaction system using High-Fidelity PCR Master Mix (NEB) and the procedure was as follows: initial denaturation at 98°C for 1 min; 98°C for 10 s, 60°C for 30 s, and 72°C for 30 s, for 35 cycles; and a final extension at 72°C for 5 min. The PCR products were visualized by 1.5% agarose gel electrophoresis analysis.

### Identification and Phylogenetic Analysis of the *Echinococcus* Venom-Allergen-Like Protein (VAL) Gene Family

Using protein sequences of *Schistosoma mansoni* VALs (Chalmers et al., 2008) as query sequences, the initial protein sequences of *Echinococcus* VALs were retrieved from the non-redundant protein sequence database of the National Center for Biotechnology Information by BLASTP search (*E*-value cut-off: 10^-5^). CD-HIT v4.5.4 software^1^ was then used to eliminate redundant protein sequences. All remaining protein sequences were further examined for the presence of SCP/TAPS-representative protein domains by searching the Conserved Domain Database (Marchler-Bauer et al., 2011) of the National Center for Biotechnology Information. A phylogenetic relationship tree was built using the full-length amino acid sequences of *Echinococcus* VALs in the following steps. First, sequences were aligned using ClustalX (Larkin et al., 2007), then they were refined manually, and a phylogenetic tree was finally generated using MEGA 5.0 software (Tamura et al., 2011) by the neighbor-joining method (the bootstrap test was performed with 1000 replicates).

## Results

### Transcriptome Sequencing and Assembly

In this study, a total of 43,509,848 and 44,379,588 high-quality reads (100 bp/read) were obtained from worms of *E. granulosus* and *E. multilocularis*, respectively. Low-quality sequences were removed by a Perl program, and the retained high-quality reads were mapped to the published genomes of *E. granulosus* and *E. multilocularis* to construct unique transcript sequences. A total of 34,717,856 reads (79.79%) mapped to the *E. granulosus* genome reported by Tsai et al. (2013), 31,156,736 reads (71.61%) mapped to the *E. granulosus* genome reported by Zheng et al. (2013), and 38,882,179 reads (87.61%) mapped to the *E. multilocularis* genome (Tsai et al., 2013) (**Table 1**). Therefore, all the subsequent assemblies of reads and bioinformatics analyses, such as gene annotation and AS events, were based on the first mapping results for *E. granulosus*. Using the software StringTie, 24,550 (7,925 known and 16,625 novel transcripts) and 23,771 transcripts (8,432 known and 15,339 novel transcripts) were assembled for *E. granulosus* and *E. multilocularis*, respectively, and the assembly yielded 11,330 genes (6,815 known and 4,515 novel genes) for *E. granulosus* and 10,101 genes (7,051 known and 3,050 novel genes) for *E. multilocularis* (**Table 1** and Supplementary Tables S2, S3), compared with the reference genome data. The mean length of transcripts for the two parasites was 2016 and 2086 bp, respectively (**Table 1**). All sequence data has been deposited in the GEO database^2^ with an accession number of GSE59173.

**Table 1:** Summary data of the transcriptome analysis.

Class	*E. granulosus*	*E. multilocularis*
Number of single reads	43,509,848	44,379,588
Number of reads and percentages mapped to genome	34,717,856 (79.79%)^a^	38,882,179 (87.61%)
	31,156,736 (71.61%)^b^	
Assembled genes (loci)	11,330	10,101
Known	6,815	7,051
Novel	4,515	3,050
Assembled transcripts	24,550	23,771
Known	7,925	8,432
Novel	16,625	15,339
Mean length of transcripts	2,016	2,087

^a^*Number of reads and percentage mapped to published *E. granulosus* genome by Tsai et al. (2013). ^b^Number of reads and percentage mapped to published *E. granulosus* genome by Zheng et al. (2013)*.

**FIGURE 1:**
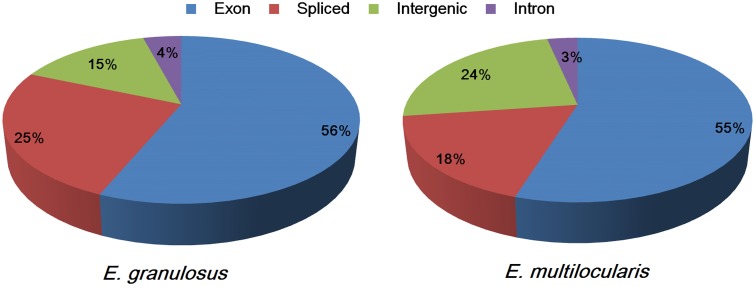
**Proportions of the four types of sequence reads mapping to the genomes of *E. granulosus* and *E. multilocularis***. The percentages of exon (the highest) and intron (the lowest) sequences were similar between the two parasites, whereas the percentages of spliced and intergenic sequences were significantly different between the two parasites.

The sequence reads could be classified into four types: exon, intron, intergenic, and spliced. The proportions of the four sequence types in the *E. granulosus* transcriptome were 56% (exons), 4% (introns), 15% (intergenic), and 25% (spliced) (**Figure 1**). The percentages of exons and introns in the *E. multilocularis* transcriptome were 55 and 3%, which is similar to the *E. granulosus* transcriptome; while the proportions of intergenic and spliced transcripts in the *E. multilocularis* transcriptome were 24 and 18%, which is significantly different from the proportions in the *E. granulosus* transcriptome (**Figure 1**). In our transcriptome database, the total exon lengths for *E. granulosus* and *E. multilocularis* were 22,249,494 bp (19.80% of the genome) and 21,805,251 bp (19.17% of the genome), which were longer than the total exon lengths in the reference genome databases for *E. granulosus* (15,213,358 bp, 13.54%) and *E. multilocularis* (16,112,293 bp, 14.16%) (Tsai et al., 2013).

### A Large Number of AS Events Were Identified in *E. granulosus* and *E. multilocularis*

**FIGURE 2:**
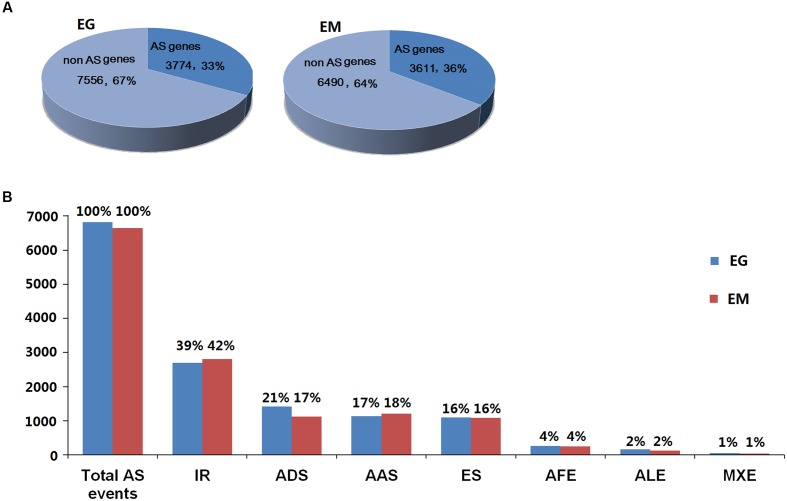
**General information about AS events in *E. granulosus* and *E. multilocularis*. (A)** Prevalence of AS in protoscolex transcriptomes of the two parasites. **(B)** Proportions of the seven types of AS in protoscolex transcriptomes of the two parasites. EG, *E. granulosus*; EM, *E. multilocularis*; IR, intron retention; ADS, alternative donor site; AAS, alternative acceptor site; ES, exon skipping; AFE, alternative first exon; ALE, alternative last exon; MXE, mutually exclusive exon.

A total of 6,826 AS events from 3,774 *E. granulosus* genes (33.31%) and 6,644 AS events in 3,611 *E. multilocularis* genes (35.75%) were bioinformatically predicted (**Figure 2A** and Supplementary Tables S4, S5). These AS events could be classified into seven types: intron retention, exon skipping, alternative donor site, alternative acceptor site, alternative first exon, alternative last exon, and mutually exclusive exon. The results showed that the proportions of the seven AS types were similar in *E. granulosus* and *E. multilocularis*. Intron retention (39 and 42%) was the most common AS type, followed by alternative donor site (21 and 17%), alternative acceptor site (17 and 18%), exon skipping (both 16%), and the least common splicing forms were alternative first exon (both 4%), alternative last exon (both 2%), and mutually exclusive exon (both 1%) in the two parasites, *E. granulosus* and *E. multilocularis*, respectively (**Figure 2B** and Supplementary Tables S4, S5).

### A Panel of AS Events Were Validated by RT-PCR

**FIGURE 3:**
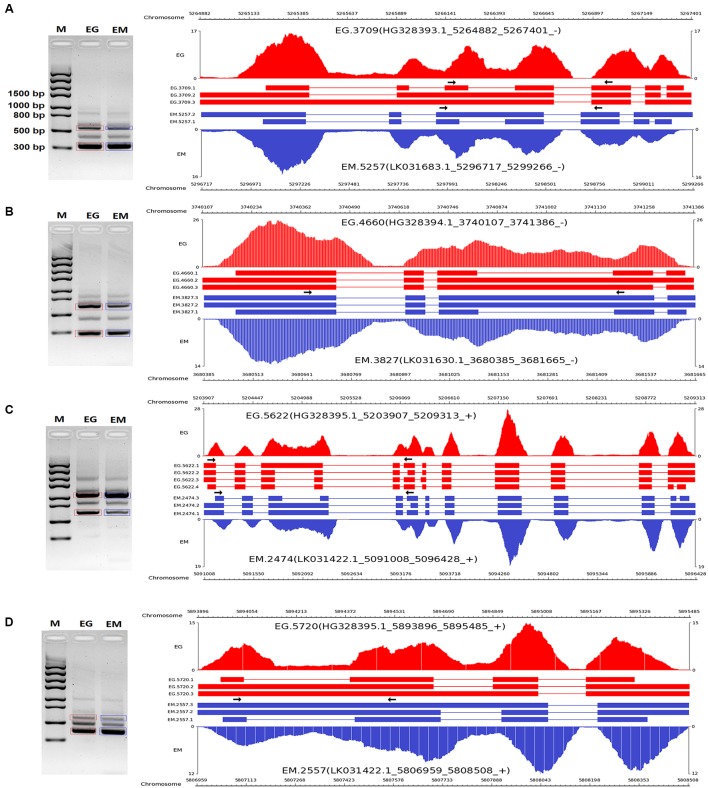
**RT-PCR validation of intron-retention events in *E. granulosus* and *E. multilocularis***. A subset of intron-retention events **(A–D)** in protoscoleces were confirmed by RT-PCR. Paired arrows in the gene frames indicate where primers were designed for each AS event. RT-PCR products were visualized with 1.5% agarose gels. Electrophoretic bands corresponding with predicted AS events in *E. granulosus* and *E. multilocularis* are marked by red and blue boxes, respectively. M, marker; EG, *E. granulosus*; EM, *E. multilocularis*.

**FIGURE 4:**
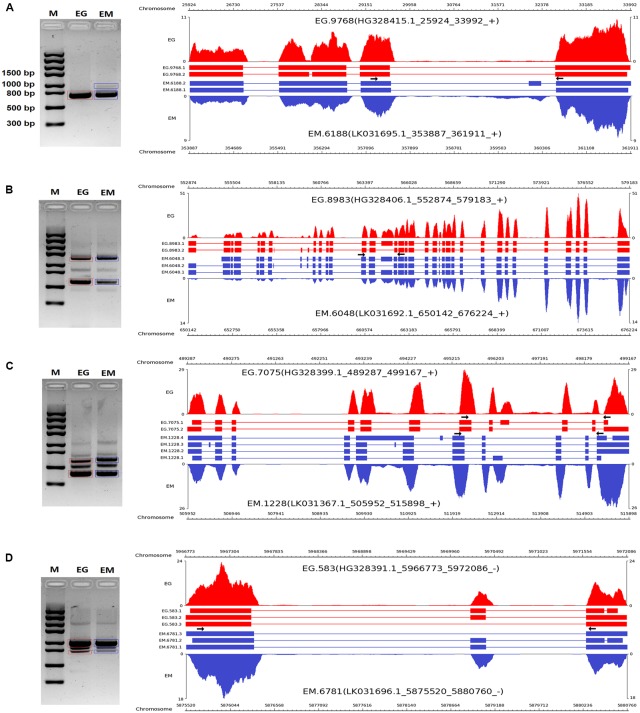
**RT-PCR validation of exon skipping events in *E. granulosus* and *E. multilocularis***. A subset of four exon skipping events **(A–D)** in protoscoleces were confirmed by RT-PCR. Paired arrows in the gene frames indicate where primers were designed for each AS event. RT-PCR products were visualized with 1.5% agarose gels. Electrophoretic bands corresponding with predicted AS events in *E. granulosus* and *E. multilocularis* are marked by red and blue boxes, respectively. M, marker; EG, *E. granulosus*; EM, *E. multilocularis*.

To validate the AS events predicted by bioinformatic analysis, RT-PCR was carried out on eight pairs of homologous genes producing different types of AS events in the two parasites. Four pairs of homologous genes with intron-retention events were serine/arginine-rich splicing factor (EG.4660 and EM.3827), SNARE protein Sec22 (EG.5720 and EM.2557), hypothetical protein (EG.3709 and EM.5622), and hypothetical protein (EG.5720 and EM.2557). The other four pairs of homologous genes with exon skipping events were peroxidasin (EG.9768 and EM.6188), filamin (EG.8983 and EM.6048), adducin-related protein (EG.7075 and EM.1228), and heat shock protein 70 (EG.583 and EM.6781). Agarose gel electrophoresis showed that bands corresponding with the predicted AS events could be detected in the RT-PCR products (**Figures 3**, **4**). Notably, some extra bands detected in the RT-PCR products were proved to be additional AS events from the corresponding genes by Sanger sequencing method (**Figures 3**, **4**). The results were indicative that the bioinformatic prediction based on the primary sequencing data was reliable, and additional AS events for some genes, which could not be sequenced nor predicted in the present study, also occurred in the two parasites.

### GO and KEGG Pathway Analyses of AS Genes

**FIGURE 5:**
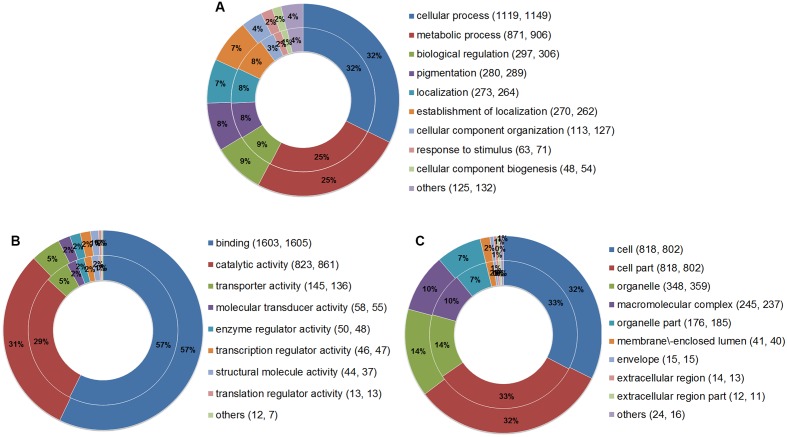
**Comparative GO analysis of genes with AS events between *E. granulosus* and *E. multilocularis***. GO terms were summarized according to three categories: biological process **(A)**, molecular function **(B)**, and cellular component **(C)**. The outer ring represents AS genes of *E. multilocularis*, and the inner ring represents AS genes of *E. granulosus*. Numbers in brackets represent the AS genes of *E. granulosus* and *E. multilocularis* falling into the corresponding GO terms.

Gene Ontology analysis was performed to summarize and explore the functional categories of the genes that underwent AS events in the two parasites. A total of 2,364 AS genes of *E. granulosus* and 2,376 AS genes of *E. multilocularis* were annotated for different GO terms (Supplementary Tables S3, S4). The results showed that the proportions of enriched GO terms for AS genes were globally similar between *E. granulosus* and *E. multilocularis* based on biological process (**Figure 5A**), molecular function (**Figure 5B**), and cellular component (**Figure 5C**). For biological processes, genes with AS events were predominantly enriched in GO terms that were relevant to cellular process and metabolic process. For the molecular function category, the majority of AS genes were annotated with the term binding, followed by the term catalytic activity.

Moreover, 1,099 and 1,115 AS genes were annotated for KEGG pathways. The enrichment pathway analysis showed that these genes were significantly enriched in 26 pathways relating to cellular processes, environmental information processing, genetic information processing, metabolism, and organismal systems (*P* < 0.05, **Table 2**). For environmental information processing, the AS genes were mainly enriched in the phosphatidylinositol signaling system, cellular antigens, and Jak-STAT, VEGF, and Notch signaling pathways, suggesting that AS may play important roles in the response to host environmental stress. For metabolism, the most significantly enriched pathways were involved in purine, fatty acid, galactose, glycerolipid, or *N*-glycan metabolism or biosynthesis. For genetic information processing, the enriched AS genes were grouped mainly in basal transcription factors, RNA transport, and the mRNA surveillance pathway. For organismal systems, the significant enrichment of AS genes included those relating to development (osteoclast differentiation), the immune system (Fc epsilon RI, T cell receptor, and Toll-like receptor signaling pathway), and the endocrine system (GnRH signaling pathway) (**Table 2**). Further experimental verification is necessary to clarify the biological regulation mechanisms of these AS events in *Echinococcus* species.

**Table 2:** Pathway enrichment analysis of AS genes in *E. granulosus* and *E. multilocularis*.

Pathway ID	Description	*E. granulosus*		*P*-value	*E. multilocularis*		*P*-value
		All genes	AS genes		All genes	AS genes	
**Cellular processes**							


ko04111	Cell cycle – yeast	45	22	2.16E - 02	50	31	1.67E - 04


ko04113	Meiosis – yeast	40	21	9.36E - 03	46	25	8.56E - 03


**Environmental information processing**							
ko04070	Phosphatidylinositol signaling system	25	16	1.62E - 03	26	22	4.90E - 07
ko04630	Jak-STAT signaling pathway	12	9	3.82E - 03	11	8	1.50E - 02


ko04370	VEGF signaling pathway	22	13	1.15E - 02	23	15	4.24E - 03


ko04330	Notch signaling pathway	15	9	3.06E - 02	13	10	3.26E - 03


ko04090	Cellular antigens	29	15	3.08E - 02	28	18	2.22E - 03


**Genetic information processing**							
ko03022	Basal transcription factors	26	14	2.45E - 02	25	17	1.17E - 03
ko03015	mRNA surveillance pathway	55	30	9.30E - 04	53	31	7.20E - 04
ko03013	RNA transport	92	48	1.38E - 04	87	47	4.58E - 04
**Metabolism**							
ko00510	*N*-glycan biosynthesis	30	20	1.88E - 04	30	23	6.78E - 06
ko00562	Inositol phosphate metabolism	20	13	3.67E - 03	20	18	8.70E - 07
ko00513	Various types of *N*-glycan biosynthesis	26	18	1.92E - 04	23	17	2.47E - 04
ko00071	Fatty acid metabolism	10	7	1.95E - 02	10	7	3.09E - 02
ko00280	Leucine and isoleucine degradation	7	6	6.82E - 03	8	6	2.96E - 02
ko00052	Galactose metabolism	19	11	2.38E - 02	19	11	4.33E - 02
ko00640	Propanoate metabolism	12	9	3.82E - 03	13	9	1.56E - 02
ko00563	GPI-anchor biosynthesis	17	11	7.92E - 03	16	10	2.85E - 02
ko00061	Fatty acid biosynthesis	3	3	3.69E - 02	3	3	4.70E - 02
ko00561	Glycerolipid metabolism	18	13	8.38E - 04	20	13	8.04E - 03
ko00230	Purine metabolism	84	41	2.26E - 03	86	41	1.77E - 02
**Organismal systems**							
ko04380	Osteoclast differentiation	17	11	7.92E - 03	16	10	2.85E - 02
ko04664	Fc epsilon RI signaling pathway	17	10	2.71E - 02	16	11	8.04E - 03
ko04912	GnRH signaling pathway	26	13	4.78E - 02	31	17	2.52E - 02
ko04660	T cell receptor signaling pathway	31	17	1.09E - 02	31	18	1.03E - 02
ko04620	Toll-like receptor signaling pathway	14	10	4.00E - 03	14	10	7.72E - 03

### Sequence Conservation Analysis of *Echinococcus* AS Genes

**FIGURE 6:**
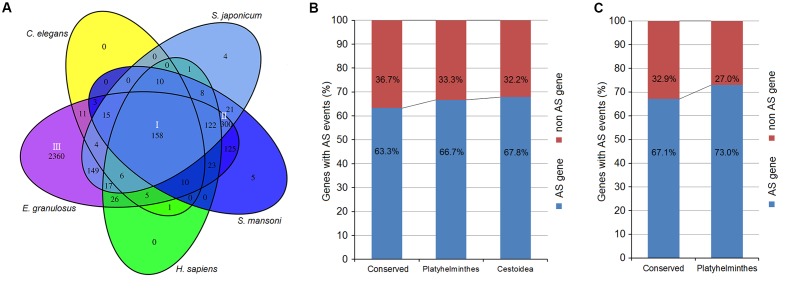
**Sequence conservation analysis of *Echinococcus* AS genes. (A)** Venn diagram of *Echinococcus* AS genes with homologous genes in the five organisms. **(B)** The percentages of *E. granulosus* genes with AS events from the three groups. **(C)** The percentages of *S. japonicum* genes with AS events from groups I and II.

Using AS gene sequences of *E. multilocularis* as the query database, we performed a BLASTX search to retrieve homologous genes in the following five organisms: *E*. *granulosus*, *S*. *japonicum, S*. *mansoni, Caenorhabditis elegan*, and *Homo sapiens*. A total of 3389 of *E. multilocularis* AS genes were identified as having homologous genes in the five organisms. Venn diagram analysis showed that there were three major groups of *E. multilocularis* AS genes: group I (conserved genes, 158 genes) (**Figure 6A** and Supplementary Table S6), for which homologous genes could be retrieved in all five organisms; group II (Platyhelminthes genes, 300 genes) (**Figure 6A** and Supplementary Table S7), for which homologous genes could only be retrieved in *E. granulosus*, *S. japonicum*, and *S. mansoni*; and group III (Cestoidea genes, 2360 genes) (**Figure 6A** and Supplementary Table S8), for which homologous genes could only be retrieved in *E*. *granulosus*. Moreover, the percentages of *E. granulosus* genes with AS events from the three groups were compared to investigate whether AS events were related to their gene sequence conservation. In group I, 63.3% of *E. granulosus* homologs with AS events were identified, which was lower than the percentages for group II (66.7%), and group III (67.8%) (**Figure 6B**). Meanwhile, we compared the proportions of *S. japonicum* genes with AS events from group I and group II using our published *S. japonicum* transcriptome data (Piao et al., 2014). The percentage of *S. japonicum* homologs with AS events in group I (67.1%) was lower than the percentage in group II (73.0%) (**Figure 6C**). The representative homologous gene pairs with AS events from the three groups are listed in **Table 3**.

**Table 3:** Representative homologous gene pairs with AS events from groups I, II, and III.

				AS								
Group	EG^a^ gene	EM gene	Annotation	AAS	ADS	AFE	ALE	MXE	IR	ES
I	EG.664	EM.6854	Serine/threonine protein kinase						+/•^b^	
	EG.5998	EM.9833	Similar to β-tubulin	+	+/•	+/•			+/•	•
	EG.7097	EM.1247	Dynamin	+	+/•				+/•	+/•
	EG.3316	EM.4881	Vacuolar proton ATPases	+/•	+			+	+/•	•
	EG.1088	EM.7236	Pre-mRNA-splicing factor CLF1		•				+	
	EG.665	EM.6855	Phospholipase C	+	+			+/•	+	+/•
	EG.5106	EM.4247	Multiple ankyrin repeats single KH domain protein	+/•					•	+/•
	EG.5383	EM.2230	*N*-Acetyltransferase 10	+			•		+/•	
	EG.3284	EM.4849	Developmentally regulated GTP-binding protein 2		+				+/•	
	EG.9830	EM.6246	Eukaryotic translation initiation factor 2c		+				+/•	
II	EG.9768	EM.6188	Peroxidasin						+	•
	EG.8983	EM.6048	Filamin							+/•
	EG.7075	EM.1228	Adducin-related protein						•	+/•
	EG.4660	EM.3827	Serine/arginine rich splicing factor						+/•	
	EG.5720	EM.2557	SNARE protein Sec22						+/•	
	EG.2713	EM.8982	Voltage-dependent calcium channel	+/•	+/•	•			+/•	+/•
	EG.215	EM.6429	Hypothetical protein		+/•					
	EG.3215	EM.9455	Aspartyl aminopeptidase	•		+			+/•	+/•
	EG.8105	EM.327	Venom allergen-like protein		+				+/•	
	EG.4029	EM.5528	Hypothetical protein			+/•		+/•		•
III	EG.583	EM.6781	Heat shock protein 70							+/•
	EG.10583	EM.149	Acyl-CoA dehydrogenase 9		+/•				+/•	
	EG.3709	EM.5622	Hypothetical protein						+/•	
	EG.5622	EM2474	Hypothetical protein		+/•				+/•	
	EG.1536	EM7652	Hypothetical protein						+	+/•
	EG.8356	EM581	Tegument antigen						•	+/•
	EG.7014	EM.1953	PDZ domain-containing protein GIPC3-like		•				+	+/•
	EG.1931	EM.8226	Hypothetical protein	+/•	+/•	+		•	+/•	+/•
	EG.2541	EM.8817	Transmembrane protein 144		•				+/•	+
	EG.2553	EM.8828	Tetraspanin		+				•	

^a^*EG, *E. granulosus*; EM, *E. multilocularis*; AAS, alternative acceptor site; ADS, alternative donor site; AFE, alternative first exon; ALE, alternative last exon; MXE, mutually exclusive exon; IR, intron retention; ES, exon skipping. ^b^+ represents an AS event in *E. granulosus* and • represents an AS event in E. multilocularis*.

### Phylogenetic Relationship and AS Analyses of the *Echinococcus* VAL Gene Family

**FIGURE 7:**
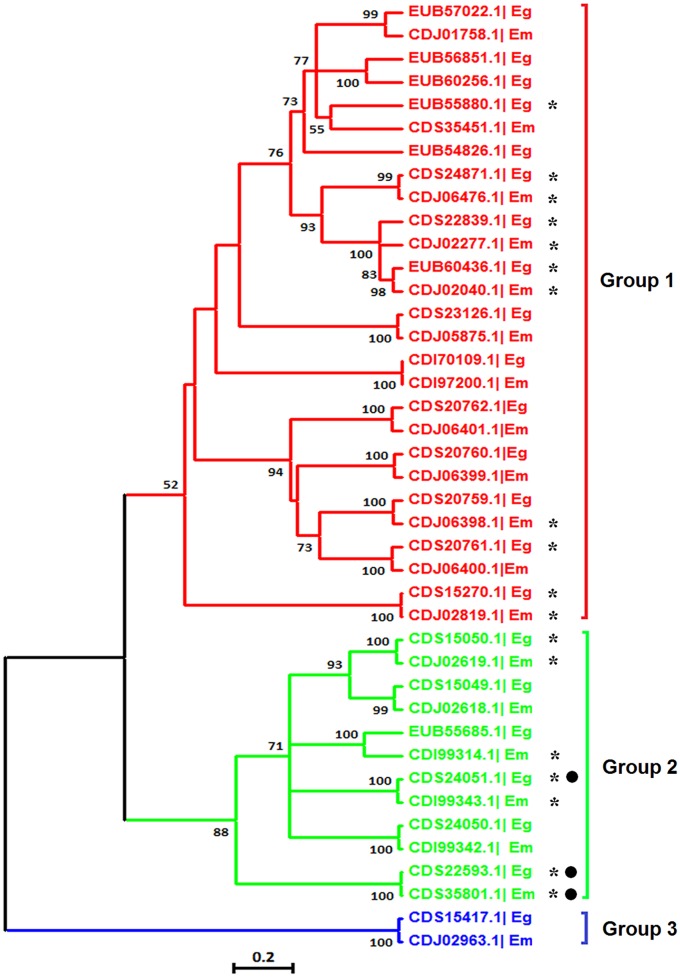
**Molecular phylogenetic relationship of the VAL gene family of *E. granulosus* and *E. multilocularis***. The tree was divided into three phylogenetic groups and the bootstrap values (more than 50) are shown at the nodes. Asterisks represent genes transcribed in protoscoleces and black dots represent genes with AS. Eg, *E. granulosus*; Em, *E. multilocularis*.

The VAL genes are members of the sperm-coating protein/Tpx-1/Ag5/PR-1/Sc7 (SCP/TAPS) superfamily, which may be important for chronic host/parasite interactions (Chalmers et al., 2008; Farias et al., 2012). It has been found that members of the VAL family exhibit multiple expression patterns and AS in the life cycle of *S. mansoni* (Chalmers et al., 2008) and *S. japonicum* (Liu et al., 2015). Based on the genome databases of the two species, we identified 22 *E. granulosus* VALs and 19 *E. multilocularis* VALs with complete SCP/TAPS domains. Next, a phylogenetic tree was reconstructed to examine the phylogenetic relationships among VALs in the two *Echinococcus* species. The result showed that the *Echinococcus* VAL family was divided into three major groups, and the majority of genes had homologous counterparts between the two *Echinococcus* species (**Figure 7**). Using these nucleotide sequences of *Echinococcus* VALs as query sequences, local BLASTN searches were performed to identify the corresponding mRNA sequences in our RNA-Seq data. Nine *E. granulosus* VALs and nine *E. multilocularis* VALs were proved to be transcribed in protoscoleces (**Figure 7**), and the others may be expressed in other developmental stages or the amount of their transcription was too low to be detected by our study. Interestingly, AS transcripts of *E. granulosus* VALs (CDS24051.1 and CDS22593.1) and the *E. multilocularis* VAL (CDS35801.1) from group II were discovered in our RNA-Seq data (**Figure 7**). Therefore, AS events may be ubiquitous in the VAL family of parasitic platyhelminths, and is well worth further study.

## Discussion

As one of major fields of enquiry in the post-genomic era, AS has been investigated in an increasing number of species over the past decade (Kalsotra and Cooper, 2011; Filichkin et al., 2015; Lunghi et al., 2016). It has been shown that AS plays a crucial role in enhancing transcriptomic and proteomic diversity, and its prevalence and characteristics vary considerably at many different levels, such as in species, tissues, and developmental stage (Kornblihtt et al., 2013). In this study, to the best of our knowledge, we have presented for the first time, a genome-wide analysis of AS events in protoscolex transcriptomes of *E. granulosus* and *E. multilocularis.* We found that approximately 33–36% of genes in the two *Echinococcus* species underwent AS events. This result is close to the AS percentage in *S. japonicum* (35%) (Piao et al., 2014) and *Drosophila melanogaster* (31%) (Graveley et al., 2011), and higher than the percentages in *Danio rerio* (17%) (Lu et al., 2010) and *C. elegans* (25%) (Ramani et al., 2011), but far below the percentage in *H. sapiens* (over 90%) (Pan et al., 2008; Wang et al., 2008). It is known that AS within the same species can be affected by developmental stage, tissue type, and growth conditions. Indeed, a higher AS percentage was obtained in schistosomulum and adult stages of *S. japonicum* (Wang et al., 2015), compared with the AS percentage in the adult stage only (Piao et al., 2014). Our estimate of AS events in *Echinococcus* species could be underestimated, since the parasite experiences several distinct environments during its complex life cycle, yet we only sequenced samples for one stage. We would expect a higher AS percentage to be observed in *Echinococcus* species when more developmental stages (such as adult, egg, and oncosphere) are analyzed.

Alternative splicing events can be commonly classified into seven types: intron retention, exon skipping, alternative donor site, alternative acceptor site, alternative first exon, alternative last exon, and mutually exclusive exon (Black, 2003). Previous studies have revealed that exon skipping, alternative donor site, alternative acceptor site, and intron retention are the four basic AS types (Nilsen and Graveley, 2010), and the overall AS patterns vary across species, tissue types, and developmental stages (Wang et al., 2008; Gibilisco et al., 2016). Exon skipping is thought to be the most prevalent AS type in animals, whereas intron retention represents the most common AS form in plants and unicellular eukaryotes (Barbazuk et al., 2008). Unexceptionally, the four major types of AS covered the vast majority of AS events in this study. Intron retention is a process by which specific introns, or fragments thereof, remain unspliced in polyadenylated transcripts. It is noteworthy that intron retention, but not exon skipping, was the predominant AS type in protoscoleces based on our high-coverage poly(A)^+^ RNA-Seq data. The advent of new sequencing technologies and algorithms is providing growing evidence that intron retention is a universal mechanism common to many eukaryotic organisms (Lunghi et al., 2016). By applying a new pipeline for intron-retention detection to high-coverage poly(A)^+^ RNA-Seq data, Braunschweig et al. (2014) found that intron retention is far more frequent in mammals than previously believed, affecting transcripts from as many as three-quarters of intron-containing genes. Meanwhile, previous studies have shown that intron retention is developmentally regulated and specific to the developmental phase or tissue (Boothby et al., 2013; Wang et al., 2016). In a study by Dvinge and Bradley (2015), it was found that an increase in retained introns is common in most cancer transcriptomes affecting genes involved in RNA processing and nuclear export. Moreover, the identification of increasing numbers of regulated intron-retention events in multi-stage unicellular parasites and plants suggests that these mechanisms can facilitate adaptation to acute host environmental changes, or duly supply key molecules to invade and exploit different ecological niches (Filichkin et al., 2015; Lunghi et al., 2016). The amazingly high frequency of intron retention in protoscoleces of *Echinococcus* species revealed that intron retention may play crucial roles in the development of the parasite and in host–parasite interaction. It will be fascinating to investigate the diversity of AS occurring in different developmental stages of *Echinococcus* species in the future. Deeper mechanistic research of intron retention will provide novel insight into its role in regulating gene expression during the life cycle of the parasites. Intron-retention mechanisms deserve to be elevated from paltry biological events to key elements that facilitate the functional tuning of cells, tissues or even developmental stages of numerous eukaryotes, especially for animals.

Gene Ontology analysis was performed to summarize and explore the functional categories of AS genes identified in this study. The results indicated that the proportions of enriched GO categories between the two parasites were generally consistent with each other in this study. Meanwhile, the KEGG pathway enrichment analysis showed that these AS genes tended to be enriched in multiple pathways, such as numerous metabolic pathways (e.g., purine, fatty acid, galactose, and glycerolipid metabolism) and key signaling pathways (e.g., Jak-STAT, VEGF, Notch, and GnRH signaling pathways). Interestingly, we found that 30 genes that underwent AS events were significantly enriched in mRNA surveillance pathways (*P* < 0.001), which are utilized by organisms to ensure the quality and fidelity of mRNA. As one of the well-known mRNA surveillance pathways (Lykke-Andersen and Jensen, 2015), the NMD pathway not only targets mRNAs containing premature termination codons for degradation, but also plays a key role in the regulation of physiological gene expression (Chang et al., 2007; Fatscher et al., 2015; Hug et al., 2016). Studies by Filichkin et al. (2010) and Sorber et al. (2011) are suggestive that a proportion of premature termination codon-containing transcripts generated by AS events may either produce truncated protein isoforms or tune gene expression post-transcriptionally by the NMD pathway. Although this highly conserved pathway has been reported in numerous eukaryotes, the NMD pathway has yet to be shown in *Echinococcus* species. Using human core members (UPF1, UPF2, and UPF3) of the NMD pathway as query sequences, we identified homologs to all three in the genomes of *E. granulosus* (CDS17643.1, CDS16560.1, and CDS18511.1) (Tsai et al., 2013; Zheng et al., 2013) and *E. multilocularis* (CDS42690.1, CDS41342.1, and CUT99229.1) (Tsai et al., 2013), suggesting the NMD pathway exists in *Echinococcus* species.

## Conclusion

We have performed what is, to the best of our knowledge, the first genome-wide survey of AS events in *Echinococcus* species using a next-generation sequencing technology. We found that the AS prevalence was about 33–36%, and intron retention was the predominant AS type in the protoscolex transcriptomes of *E. granulosus* and *E. multilocularis*. Genes that underwent AS events were significantly enriched in dozens of pathways mainly related to metabolism, signal transduction, and genetic information processing. These results will not only significantly improve the re-annotation of *Echinococcus* genomes, but also provide an invaluable resource for future functional and evolutionary studies of AS in the platyhelminth parasites.

## Author Contributions

Experiments were conceived and designed by SL and QC. Experiments were performed by XZ, SL, LH, XP, and NH. Data was analyzed by SL and XZ. The paper was written by SL, XZ, and QC.

## Conflict of Interest Statement

The authors declare that the research was conducted in the absence of any commercial or financial relationships that could be construed as a potential conflict of interest.

## Footnotes

1 http://www.bioinformatics.org/cd-hit/

2 http://www.ncbi.nlm.nih.gov/geo/info/linking.html

## Abbreviations

AS: alternative splicing
GO: Gene Ontology
KEGG: Kyoto Encyclopedia of Genes and Genomes
NMD: nonsense-mediated mRNA decay
RNA-Seq: RNA-sequencing.
